# Mental Condition of the King of Prussia

**Published:** 1863-08

**Authors:** 


MENTAL CONDITION OF THE KING OF PRUSSIA.—A private communication from Berlin says:—"You may judge of the King's state of mind when I tell you that, some little time ago, His Majesty was possessed with the idea that a gallows, intended for himself, was being erected under the windows of his palace. Sitting at the window, and looking out upon the court-yard, he would repeatedly say, 'They're building it, they're building it!' I need scarcely add, that nothing was being erected there."

